# Quest for Quality in Translational Stroke Research—A New Dawn for Neuroprotection?

**DOI:** 10.3390/ijms23105381

**Published:** 2022-05-11

**Authors:** Matteo Haupt, Stefan T. Gerner, Mathias Bähr, Thorsten R. Doeppner

**Affiliations:** 1 Department of Neurology, University of Goettingen Medical School, 37075 Goettingen, Germany; mbaehr@gwdg.de; 2 Department of Neurology, University Hospital Giessen, 35394 Giessen, Germany; stefan.gerner@neuro.med.uni-giessen.de; 3 Department of Anatomy and Cell Biology, Medical University of Varna, 9002 Varna, Bulgaria; 4 Research Institute for Health Sciences and Technologies (SABITA), Medipol University Istanbul, Istanbul 34810, Turkey

**Keywords:** cerebral ischemia, neuroprotection, neurodegeneration, stroke

## Abstract

Despite tremendous progress in modern-day stroke therapy, ischemic stroke remains a disease associated with a high socioeconomic burden in industrialized countries. In light of demographic change, these health care costs are expected to increase even further. The current causal therapeutic treatment paradigms focus on successful thrombolysis or thrombectomy, but only a fraction of patients qualify for these recanalization therapies because of therapeutic time window restrictions or contraindications. Hence, adjuvant therapeutic concepts such as neuroprotection are urgently needed. A bench-to-bedside transfer of neuroprotective approaches under stroke conditions, however, has not been established after more than twenty years of research, albeit a great many data have demonstrated several neuroprotective drugs to be effective in preclinical stroke settings. Prominent examples of substances supported by extensive preclinical evidence but which failed clinical trials are tirilazad and disodium 2,4-sulphophenyl-N-tert-butylnitrone (NXY-059). The NXY-059 trial, for instance, was retrospectively shown to have a seriously weak study design, a trial of insufficient quality and a poor statistical analysis, although it initially met the recommendations of the STAIR committee. In light of currently ongoing novel neuroprotective stroke trials, such as ESCAPE-NA, and to avoid the mistakes made in the past, an improvement in study quality in the field of stroke neuroprotection is urgently needed. In the present review, animal models closely reflecting the “typical” stroke patient, occlusion techniques and the appropriate choice of time windows are discussed. In this context, the STAIR recommendations could provide a useful orientation. Taking all of this into account, a new dawn for neuroprotection might be possible.

## 1. Introduction

Stroke is one of the leading causes of death worldwide [1]. The prevalence of stroke was 101 million cases in 2019, and the incident cases were 12.2 million [2]. In the same year, 6.55 million deaths and 143 million disability-adjusted life-years (DALYs) were attributed to stroke [2]. From 1990 to 2019, the total number of prevalent strokes increased by 85%, incident strokes increased by 70%, deaths from stroke increased by 43%, and DALYs due to stroke increased by 32% [2]. In 2019, 62.4% of all strokes were of ischemic etiology [2]. However, in addition to common revascularization treatments, an adjuvant therapy for ischemic stroke is lacking. Hence, several preclinical studies investigating a large number of neuroprotective substances were performed in the last few decades. The neuroprotective strategy, applied alone or in combination, aims at directly inhibiting ischemia-induced cell injury or even cell death [3]. Indeed, numerous substances were shown to have such protective effects in preclinical stroke models at acute, subacute or even delayed time points of the disease. Nevertheless, translational approaches failed until recently. In response, the Stroke Therapy Academic Industry Roundtable (STAIR) published recommendations in order to improve the quality of preclinical stroke studies [4,5]. These criteria included recommendations regarding time windows, reproducibility, animal models, drug dosing, among others. Such recommendations, however, did not result in a paradigm shift in preclinical stroke research, and additional research resources were spent in vain without a successful translational outcome.

In this review, we will first give a brief overview of the pathophysiology of stroke along with preclinical and clinical studies. Thereafter, we will discuss the challenges for bench-to-bedside translation and indicate possible reasons for failure in the past.

## 2. Pathophysiology of Stroke

To identify possible neuroprotective agents, a deep understanding of the complex pathophysiology of ischemic stroke is mandatory. In ischemic stroke, impaired perfusion results in an acute glucose and oxygen deficiency, which leads to insufficient anerobic adenosine triphosphate (ATP) production, followed by lactate acidosis and disturbed cellular homeostasis [6]. The latter causes loss of membrane potential with cellular depolarization and the opening of voltage gated Ca^2+^, Na^+^ and K^+^ channels, yielding an inadequate Ca^2+^ influx and glutamate-triggered extracellular excitotoxicity [7]. The enhanced intracellular Ca^2+^ concentration activates proteases, phospholipases and reactive oxygen species, degrading essential cellular components, such as membrane proteins, cellular lipids and nucleic acids [6]. In parallel, enhanced intracellular Na^+^ influx causes cytotoxic edema.

Apart from excitotoxicity, hypoxia-triggered inflammation is another key factor in stroke pathophysiology [6]. Minutes after hypoxia, neurons release damage-associated molecular patterns which activate resident microglia [8]. Activated microglia, in turn, migrate to the infarct core and to the penumbra and mediate detrimental or protective effects depending on their specific subtype [8]. Furthermore, the secretion of different pro-inflammatory proteins such as tumor necrosis factor α and interleukins by microglia, neurons and endothelial cells additionally amplifies inflammatory response [8]. The latter enhances the expression of adhesion molecules on endothelial cells and a breakdown of the blood–brain barrier, resulting in extravasation of immune cells into the brain parenchyma [9]. During the first minutes and hours, mainly neutrophils and monocytes enter the brain and exacerbate the inflammatory response [9]. Additionally, T cells can enter the parenchyma and exert detrimental or protective effects [9], thus contributing to neuronal cell loss, mainly through necrosis or apoptosis, although additional and novel forms of cell death are likely to be involved as well.

**Figure 1:**
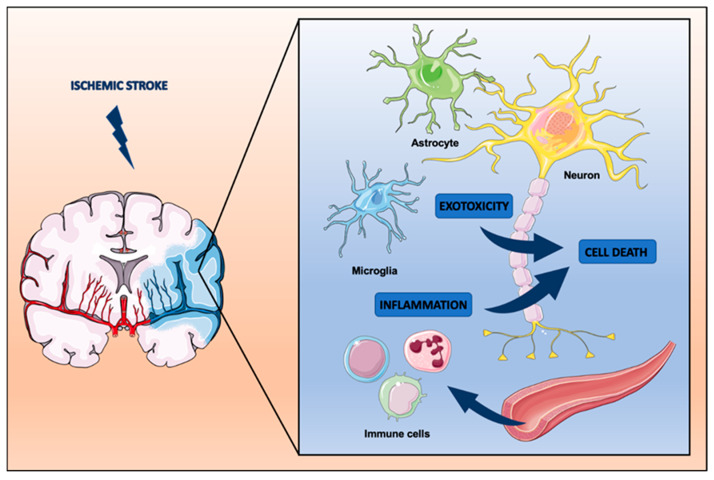
Pathophysiology of ischemic stroke and common targets for neuroprotection.

As a matter of fact, the pathophysiology of ischemic stroke, as briefly outlined, offers a variety of therapeutic targets that range from excitotoxicity to inflammation and various forms of cell death. Elucidating appropriate biologically active agents that may interfere with these signaling cascades constitutes the central strategy for neuroprotective stroke research to this day (Figure 1).

## 3. Preclinical Studies—A Brief Overview

The majority of preclinical stroke studies focus on the acute phase of ischemic stroke. Apart from models of hypoxia with combined oxygen and glucose deprivation (OGD), stroke models with rodents are abundant. The different occlusion techniques to induce cerebral ischemia in these animals are discussed in detail in a later section of this review. Focusing on the acute time window of the disease, a great many studies make use of potentially neuroprotective drugs within six hours after stroke onset. Drugs or biologically active agents that do not cross the blood–brain barrier, e.g., mesenchymal stem cells, are occasionally delivered stereotactically as well [10].

Neuroprotective agents can be classified into major subgroups, among which are free-radical scavengers, glutamate antagonists and anti-inflammatory agents [11]. In addition, stem cells, such as mesenchymal stem cells (MSCs) and stem cell-derived extracellular vesicles, have shown promising preclinical effects [12], although their mode of action appears to focus beyond the subacute time point of stroke. Such preclinical strategies are therefore beyond the scope of the present review, which focuses on (sub-)acute neuroprotection.

The application of free radical scavengers—citicoline or NXY-059, for instance—has been shown to provide substantial neuroprotective effects in preclinical studies but has failed in terms of clinical translation [13,14,15]. Nerinetide is a neuroprotective eicosapeptide which blocks pathological activation of neuronal NO synthetase by inhibition of the NMDAR–postsynaptic density protein (PSD 95) interaction [16]. Its intravenous application resulted in reduced stroke injury, as shown in cultured neurons, rodents and primates [16,17,18]. Similarly, NSP-116, another free radical scavenger, also shows neuroprotective effects under conditions of both preclinical ischemic and hemorrhagic stroke, such that it might be of interest in future clinical trials [19]. In this context, the NMDA receptor antagonist MK801 has been investigated in over 50 preclinical studies where it was found to effectively reduce brain lesion volume and the extent of cerebral edema in stroke rodents [20]. A clinical trial, however, is missing to date. Other glutamate antagonists, such as selfotel, aptiganel and gavestinel, have also showed protective effects in preclinical studies but have proved to be not efficacious in patients with acute ischemic stroke [21,22].

Anti-inflammatory drugs targeting different steps in neuroinflammation, such as microglial activation, interleukin secretion, and immune cell infiltration, have also been repeatedly reported. Of note, these substances usually do not exclusively regulate one single inflammatory pathway but have pleiotropic effects instead. Such drugs of interest include tocilizumab, fasudil and veliparib [23,24,25]. Furthermore, many preclinical studies have revealed that transplanted MSCs may modulate post-ischemic immune responses, although such mechanisms imply neurogenesis and angiogenesis rather than genuine neuroprotection [26]. In terms of biological signaling cascades, MSCs appear to mediate neuroprotection or neuroregeneration by partly modulating inflammation through the release of extracellular vesicles (EVs). These small membrane-encapsulated vesicles in the range of nanometers have gained increasing interest in recent years; EVs have been demonstrated to be non-inferior to their host stem cells with regard to their therapeutic potential against stroke [27]. EVs serve as cargo carriers for nucleic acids, proteins and lipids, all of which affect stroke outcomes in their own specific ways [28]. However, EV contents depend on the state of host cell functioning, which determines different metabolic patterns of secreted EVs. As such, microRNA patterns of EVs may change accordingly, and accumulation of specific compounds, such as miR-17-92 or miR-1906, can enhance the therapeutic potential of EVs even further [29,30]. The fact that EVs are able to cross the intact blood–brain barrier after systemic delivery makes them attractive candidates for future stroke therapy [31]. Robust clinical data relating to their therapeutic potential in humans, however, have not yet been generated.

## 4. Translational Stroke Studies—A History of Failure

Preclinical evidence of hundreds of substances with neuroprotective features in animal models led to translational clinical trials in some cases. Some examples of these failed bench-to-bedside translations are presented below, together with a short outline of possible reasons for failure.

In over 18 preclinical studies using 544 animals, tirilazad demonstrated promising neuroprotective effects as a free radical scavenger [32]. Tirilazad reduced infarct volumes by 29% and enhanced neurobehavioral scores by 48% on average [32]. This promising evidence resulted in a multicenter, randomized, double-blinded, vehicle-controlled trial of tirilazad mesylate in patients with acute stroke (RANTTAS) [33]. Therefore, 276 patients were treated with tirilazad and 280 patients were given a vehicle within 6 h after stroke onset for 3 days [33]. The study, however, was stopped after the results suggested that tirilazad does not improve neurological outcome as quantified using the Glascow Coma Scale and the Barthel Index [33]. A follow-up study (RANTTAS II) in which higher dosages of tirilazad were used was prematurely stopped by the sponsor when questions regarding safety emerged from a parallel study in Europe (TESS II) [34]. A systematic review investigating the outcome of all clinical trials using tirilazad (four published and two unpublished) revealed that tirilazad treatment actually worsened outcomes in cases of acute ischemic stroke [35]. The systematic review by Sena et al. indicated two major issues regarding this failed translation [32]. First, data from animal studies suggested that tirilazad was effective only in a narrow dose range. Clinical trials with tirilazad, however, used a broad range of doses. If there is only a narrow effective dose range also in humans, this might explain the negative outcomes [32]. Second, in preclinical stroke settings, the interval between stroke onset and treatment was a median of 10 min, whereas in clinical studies it was after 5 h [32]. To make things worse, the preclinical data were not homogeneous, as tirilazad was only effective in lissencephalic but not in gyrencephalic species [32].

Preclinical data for disodium 2,4-sulphophenyl-N-tert-butylnitrone (NXY-059), a free radical spin trap agent, suggested pleiotropic effects on the ischemic cascade leading to neuroprotective effects [36,37]. In contrast to tirilazad, NXY-059 was demonstrated to be neuroprotective in non-human primates, as shown in a trial fulfilling the STAIR criteria [38]. These promising preclinical data, together with evidence of safety, led to two Phase III randomized placebo-controlled trials using NXY-059 within 6 h after stroke onset [39]. In the first trial (SAINT I), a statistical difference was suggested in favor of reduced disability after NXY-059 treatment in patients with acute ischemic stroke [40]. To confirm these observations, a second, much larger, trial (SAINT II), with approximately three thousand patients suffering from acute ischemic stroke, was conducted [40]. However, the SAINT II trial did not confirm the results of the SAINT I trial. Conclusively, NXY-059 was shown to be ineffective for the treatment of acute ischemic stroke within 6 h after the onset of symptoms [40]. Subsequently, AstraZeneca, the company which sponsored the clinical trials, stopped further investigations on NXY-059 in stroke settings.

The failure of the SAINT II trial raised doubts about preclinical evidence generated in animal stroke models and finally about the general concept of neuroprotection as a therapeutic strategy in acute ischemic stroke. A systematic review by MacLeod and colleagues analyzed the preclinical data on NXY-059 in rodent stroke studies [39]. The analysis confirmed that all animal studies, considered together, fulfilled the STAIR criteria. However, there were substantial differences in the quality of individual studies [39]. The authors suggested three factors for the overstated efficacy of NXY-059, namely, publication bias, the potential existence of unpublished negative data and the fact that only one of the preclinical studies fulfilled all the STAIR criteria on its own. In addition, a substantial number of studies had methodical weaknesses, i.e., studies that did not use randomization reported a higher efficacy than studies of higher methodological quality [39]. The authors concluded that the convincing preclinical efficacy was mainly confounded by low study design quality [39]. In line with this, only a minority of studies measured cerebral blood flow during middle cerebral artery occlusion (MCAO) in order to ensure constant infarct volumes [41]. Whereas the SAINT trials used NXY-059 for up to 6 h after stroke onset, the preclinical studies determined a maximum efficacy when NXY-059 is delivered within 4 h [41]. In addition, the stroke population in the SAINT trials displayed heterogeneous stroke patterns, including lacunar and posterior strokes, which did not match the infarct pattern of MCAO animals [41]. Finally, a biodistribution analysis revealed that only small quantities of NXY-059 are able to cross the blood–brain barrier, which raised questions as to the mode of action of NXY-059 [42].

With the failure of the SAINT trials, neuroprotection against stroke appeared to have reached a dead end. Nevertheless, new concepts and strategies have risen from the aforementioned failures of translational stroke trials on neuroprotection. Such findings have led to combined therapeutic approaches, applying both recanalizing and neuroprotective strategies, as represented in the ESCAPE-NA1 trial [43]. More than 1000 patients with ischemic stroke due to large-vessel occlusion were randomized within a 12 h treatment window to receive a single dose of nerinetide (2.6 mg/kg, up to 270 mg) or placebo adjunct to endovascular thrombectomy in this multicenter Phase III trial. Of interest, the authors failed to identify patients with large ischemic penumbra by perfusion-based imaging, despite the hypothesized mechanism of action being that of a “penumbra stabilizer” [44]. The clinical results were disappointing, with similar proportions of the primary endpoint (mRS 0–2 at 90 days) among both groups and no statistical signal of beneficial effects of nerinetide in terms of clinical or radiological secondary outcomes [43]. These negative results of the main analysis seem to be due to the difficulties of translation from-bench-to-bedside. A subanalysis of patients without intravenously administered alteplase (~40%) revealed a surprisingly high benefit of nerinetide treatment, as reflected by improved functional outcomes and by a 7.5% reduction in mortality. Unexpected drug–drug interactions led to reduced plasma-levels of nerinetide in patients with alteplase-treatment and are suspected to be the reason for the lack of treatment effects in this subgroup of patients [43,45]. Further Phase III trials are ongoing to investigate (1) the in-hospital use of nerinetide in ischemic stroke patients undergoing mechanical thrombectomy without prior alteplase-treatment in the 12 h time window (ESCAPE-NEXT; NCT04462536) and (2) the prehospital administration of nerinetide in patients with suspected stroke (FRONTIER; NCT02315443). A preplanned analysis of non-thrombolyzed ischemic stroke patients of ESCAPE-NA1 and ESCAPE-NEXT will clarify whether nerinetide represents the first clinically relevant neuroprotectant for ischemic stroke patients.

## 5. Challenges and Perspectives for Bench-to-Bedside Translation

Given the fact that bench-to-bedside translation has failed to this day, possible sources of errors must be identified and eliminated. Consequently, for successful bench-to-bedside translation, aspects such as (1) the general STAIR recommendations, (2) animal models, (3) occlusion techniques, (4) as well as time windows and possible side effects, should be considered.

### 5.1. STAIR Recommendations

**Figure 2:**
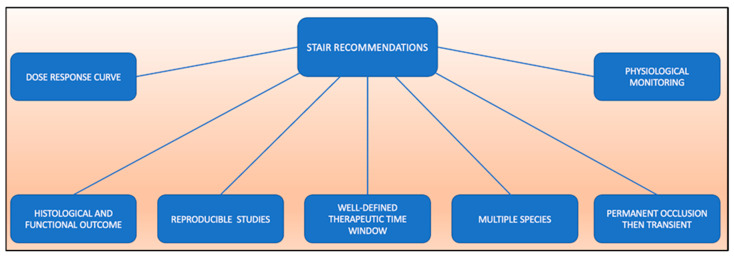
Overview of the Stroke Therapy Academic Industry Roundtable (STAIR) recommendations.

The STAIR recommendations, initially published in 1999, were intended to improve the quality of preclinical studies investigating possible novel stroke therapies [5]. According to the initial STAIR recommendations, adequate dose–response curves, defined time windows in well-characterized models, blinded and controlled reproducible studies, assessment of histological and functional outcomes, and permanent vessel occlusion followed by transient occlusion should be provided (overview in Figure 2). In addition, initial rodent studies should be followed by studies in gyrencephalic species. The aspects of dose–response curves, therapeutic time windows, outcome measures, multiple species use and reproducibility were revised in an updated version but remained valid [4]. To fulfil reproducibility, experiments should be replicated in at least one or more independent laboratory. The fundamentals of good scientific inquiry should also be applied by implementing randomization and eliminating outcome assessment bias, reporting the reasons for excluding animals from final data analyses and defining inclusion/exclusion criteria, performing power analysis and sample size calculations, and declaring any relevant conflicts of interest [4]. However, these criteria are not binding, and a vast number of studies still do not consider them fully.

### 5.2. Animal Models

Essential for a successful bench-to-bedside translation is the use of appropriate animal models. For stroke models, rats and mice are by far the most commonly used animals, although rabbits and non-human primates are becoming increasingly popular [46]. Most of the rats or mice used in stroke models are young, male animals without any typical comorbidities, such as hypercholesterinemia, arterial hypertension, diabetes or obesity. Thus, these animals are more stress-resistant and healthier in contrast to most stroke patients. This mismatch between stroke patients and animal models may affect individual outcomes significantly. However, different animal models have been introduced to better mimic stroke patients. For example, hypercholesterinemia is reflected by using apolipoprotein-E (ApoE)−/−, LDL receptor−/− or human ApoB transgenic rodents [47]. Plasma cholesterol levels can be further increased by placing rodents on a western diet [48]. Indeed, brain injury after ischemic stroke has been shown to be exacerbated in ApoE−/− mice, demonstrating the relevance of using such models [49]. Mimicking hypertension, the most prevalent risk factor for stroke [50], can be achieved by using primary hypertensive or salt-sensitive rats that display heightened immune responses poststroke, which leads to enlarged infarct volumes [51,52]. Likewise, some models induce hypertension by clipping one or both kidney arteries, resulting in systolic blood pressure values of 200–225 mmHg [53].

Diabetes increases the risk of stroke up to 3 times [54]. Models with spontaneously diabetic animals or induced diabetes are used in preclinical studies [55]. King gave an extensive overview of the different diabetes animal models in his review [56]. For example, the repeated application of Langerhans β cell toxin, which leads to a loss of β cells in the pancreas, is a widely used technique for inducing type 1 diabetes in animal models [56]. Type 2 diabetes can be triggered with a similar approach when β cell toxin is applied just once [56]. Obesity and diabetes often go hand in hand in clinical settings, and therefore animals that are deficient for leptin and the leptin receptor are an elegant way of modeling such a setting [56]. Leptin deficiency leads to hyperphagia in mice, resulting in obesity and hyperglycemia at the age of 4–8 weeks [56]. Not surprisingly, in obese and diabetic mice poststroke, long-term motor recovery is significantly impaired [57]. Future studies must therefore focus more on comorbidity models to reflect the clinical situation more adequately.

### 5.3. Occlusion Techniques

Ischemic stroke is characterized by the occlusion of brain-supplying arteries, resulting in decreased cerebral blood flow. Various stroke model techniques are used to mimic this insult in animals. Selecting an appropriate model is crucial for the significance of the results in relation to future translations. The following five techniques are most used: (1) intraluminal occlusion, (2) transcranial occlusion, (3) cerebral photothrombosis, (4) endothelin-1 occlusion and (5) cerebral embolism. Each technique has advantages and disadvantages which will be discussed in the following paragraphs (summarized in Table 1).

The most widely used experimental stroke technique is the intraluminal MCAO model [46]. In this model, a monofilament is inserted into the common carotid and then pushed forward to the internal carotid until the MCA branches off [58]. Thus, cerebral blood flow is decreased to 10–20% but rapidly restored after removal [58]. The occlusion time determines the extent of ischemic damage, which varies between 10 and 120 min [58]. This results in a large infarct core surrounded by a well-defined penumbra [59]. On account of the latter, the model is suitable for neuroprotective studies. However, to create reproduceable insults, in vivo measurement of cerebral blood flow during the occlusion is essential [59]. The lack of cerebral blood flow measurement may be one factor for false results in the past, together with the fact that such a model does not include the presence of a thrombus. Studies on thrombolysis are therefore not feasible in this model.

Using transcranial occlusion, the MCA can be occluded permanently or transiently across a cranial window [60]. Techniques such as coagulation, clip or suture find use and are often combined with ipsilateral common carotid artery occlusion [59]. On the one hand, the latter allows the generation of large brain infarcts with pronounced neurological deficits [59]. On the other hand, it is possible to produce smaller infarcts in comparison to intraluminal occlusion along with a well-defined penumbra. A potential weakness is that variants of cerebrovascular anatomy are not correctly identified, and the exact placement of MCA occlusion is insufficiently standardized [59].

The model of photothrombosis includes the systemic delivery of a photosensitive dye that is transcranial-illuminated, resulting in thrombus formation that locally occludes microvessels [61]. The advantage of this technique is the possibility of producing precise and well-defined infarcts in specific regions by stereotactic precision [61]. This model has disadvantages, too, however, since lesions are located superficially, with no genuine penumbra, and hardly react to thrombolytic drugs.

Endothelin-1 is a long-acting vasoconstrictive peptide which is administered directly to an exposed vessel, via stereotactical injection, or delivered on the cortical surface only [62]. The endothelin-1 injection results in a significant drop in cerebral blood flow that lasts over a period of hours followed by reperfusion [63]. The advantage of this model lies in its having extremely low mortality rates as a consequence of being a less invasive technique. However, since endothelin-1 has been shown to induce astrocytosis and to facilitate axonal sprouting on its own, this could lead to the misinterpretation of experiments evaluating poststroke neural repair [64].

Cerebral embolism is the most common etiology of ischemic stroke and is therefore, in respect to future clinical translation, highly relevant. There are mainly three different types of cerebral embolism models used in rodents, i.e., synthetic macrospheres or microspheres, autologous blood clots and stereotactic thrombin delivery [59]. All of these models create a well-defined penumbra and are on this account well-suited to study neuroprotective drugs administered alone as well as in combination with thrombolytic agents [62]. However, these models produce highly variable infarcts compared to intraluminal MCAO models and are associated with poor long-term animal survival [59].

### 5.4. Therapeutic Time Window

In a substantial number of preclinical neuroprotective studies, the therapeutic agents were given prior or immediately after the induction of cerebral ischemia. This does not reflect the clinical situation. As such, it is mandatory to generate evidence for drugs given within a reasonable time window if bench-to-bedside translation is desired. Neuroprotective drugs play a key role in translation, but in preclinical studies they have generally been found to have a short therapeutic time window, impairing clinical translation even further [11]. NXY-059, for example, discussed in detail above, has been shown to have a maximum therapeutic window of 4 h in preclinical studies. In clinical trials, however, patients received NXY-059 up to 6 h poststroke, which could be one explanation for the failure of the clinical trial [41].

## 6. Conclusions and Perspective

Stroke is one of the leading causes of death and disability worldwide. Hence, there is an urgent need to develop neuroprotective strategies. Ischemic stroke is characterized by a complex pathophysiology involving excitotoxicity, inflammation and various forms of cell death. Studying appropriate biologically active agents that may interfere with these signaling cascades, especially in the acute phase of stroke, constitutes the central strategy of neuroprotective stroke research, past and present. Such agents can be classified into major subgroups, among which are free-radical scavengers, glutamate antagonists and anti-inflammatory agents. In addition, stem cells such as MSCs and stem cell-derived extracellular vesicles have shown promising preclinical effects. However, despite enormous research efforts in the past, no effective therapy has been discovered.

Possible reasons for the continuing failure are statistical weaknesses in studies, non-transferable study designs and overall low study quality. Conclusively, an improvement in the quality of preclinical neuroprotection studies is needed. This requires the use of animal models reflecting the typical stroke patient, occlusion methods that properly match research questions and, finally, therapeutic times windows that are transferable to the clinical setting. Hence, the STAIR recommendations may give a useful orientation. Taking this into account, a new dawn for neuroprotection may be possible.

## Figures and Tables

**Table 1:** Overview of occlusion techniques in ischemic stroke models.

Technique	Procedure	Advantage	Disadvantage
Middle cerebral artery occlusion	– Monofilament inserted into CCA and pushed forward to ICA until MCA branches off. – Removal after defined occlusion time.	– CBF decreases rapidly and is restored after filament removal. – Large infarct with well-defined penumbra. – Well suitable for neuroprotective studies.	– CBF measurement is mandatory to create reproducible insults. – Experienced surgeon is needed to create reliable results.
Transcranial occlusion	– Coagulation, clip, or suture can be used for transient or permanent MCA occlusion across a cranial window. – Often combined with ipsilateral CCA occlusion.	– Generation of very small infarcts with good defined penumbra. – Generation of large brain infarcts with pronounced neurological deficits.	– Experienced surgeon is needed to create reliable results. – Difficult identification of cerebrovascular anatomy variants. – Insufficiently standardized placement of MCA occlusion.
Cerebral photothrombosis	– Systemic delivery of photosensitive dye that is transcranially illuminated, resulting in thrombus formation that locally occludes microvessels.	– No experienced surgeon is needed. – Possibility to produce well-defined infarcts in specific regions by stereotactic precision	– No genuine penumbra. – Hardly reacts to thrombolytic drugs.
Endothelin-1 occlusion	– Long-acting vasoconstrictor. – Administered directly to the vessel via stereotactical injection or delivered on the cortical surface.	– Extremely low mortality rates. – No experienced surgeon is needed.	– Induction of astrocytosis and axonal sprouting could lead to misinterpretation in experiments evaluating poststroke neural repair.
Cerebral embolism	– Synthetic macrospheres or microspheres, autologous blood clots, and stereotactic thrombin delivery.	– Well-defined penumbra. – Well-suited to study neuroprotective drugs only, as well as in combination with thrombolytic agents.	– Highly variable infarcts compared. – Poor long-term animal survival.

## Abbreviations

CBF	cerebral blood flow
ICA	internal carotid artery
CCA	common carotid artery
MCA	middle cerebral artery
